# The “Great Debate” at Immunotherapy Bridge 2022, Naples, November 30th–December 1st, 2022

**DOI:** 10.1186/s12967-023-04117-3

**Published:** 2023-04-22

**Authors:** Paolo A. Ascierto, Renier Brentjens, Samir N. Khleif, Kunle Odunsi, Katayoun Rezvani, Marco Ruella, Ryan J. Sullivan, Bernard A. Fox, Igor Puzanov

**Affiliations:** 1 Department of Melanoma, Cancer Immunotherapy and Innovative Therapy, Istituto Nazionale Tumori IRCCS “Fondazione G. Pascale”, Naples, Italy; 2 Department of Medicine, Roswell Park Comprehensive Cancer Center, Buffalo, NY USA; 3 The Loop Immuno Oncology Laboratory, Georgetown University Medical School, Washington, DC USA; 4 University of Chicago Medicine Comprehensive Cancer Center, Chicago, IL USA; 5 Department of Stem Cell Transplantation and Cellular Therapy, The University of Texas MD Anderson Cancer Center, Houston, TX USA; 6 Center for Cellular Immunotherapies and Division of Hematology-Oncology, University of Pennsylvania, Philadelphia, PA USA; 7 Melanoma Program, Massachusetts General Hospital Cancer Center, Boston, MA USA; 8 Earle A. Chiles Research Institute, Robert W. Franz Cancer Research Center, Providence Cancer Institute, Portland, OR USA

**Keywords:** Cancer, Immunotherapy, Adoptive cell therapy, Biomarkers, CAR therapy

## Abstract

The 2022 Immunotherapy Bridge congress (November 30–December 1, Naples, Italy) featured a Great Debate session which addressed three contemporary topics in the field of immunotherapy. The debates included counterpoint views from leading experts and considered whether adoptive cell therapy (ACT) has a role in the treatment of solid tumors, the use of peripheral/blood biomarkers versus tumor microenvironment biomarkers for cancer immunotherapy and the role of chimeric antigen receptor T cell versus natural killer cell therapy. As is the tradition in the Immunotherapy Bridge Great Debates, speakers are invited by the meeting Chairs to express one side of the assigned debate and the opinions given may not fully reflect their own personal views. Audiences voted in favour of either side of the topic both before and after each debate.

## Introduction

As is now traditional, the 2022 Immunotherapy Bridge congress (November 30–December 1, Naples, Italy) featured a Great Debate session which addressed three contemporary topics in the field of immunotherapy. The debates included counterpoint views from leading experts and considered whether adoptive cell therapy (ACT) has a role in the treatment of solid tumors, the use of peripheral/blood biomarkers versus tumor microenvironment (TME) biomarkers and, finally, the role of chimeric antigen receptor (CAR) T cell therapy versus CAR natural killer (NK) cell therapy. As is the tradition in the Immunotherapy Bridge Great Debates, speakers are invited by the meeting Chairs to express one side of the assigned debate and the opinions given may not fully reflect their own personal views. Audiences voted in favour of either side of the topic both before and after each debate.

### Cell therapy for solid tumors: yes or no?

#### Renier Brentjens: YES

Target antigen heterogeneity and immune escape are recognised problems in the use of ACT for the treatment of solid tumors, which are clearly not the same as hematologic tumors. In addition, an immune-suppressive TME in solid tumors inhibits CAR T cells and other targeted cell products. Even in the context of B-cell malignancies, current results are not optimal. However, we are only at the start of using this technology.

CAR T cell products can be designed to target multiple different tumor-associated antigens (TAAs) to overcome antigen escape or heterogeneity. One approach is dual targeted T cells. For example, CAR T-cell therapy for multiple myeloma targeting B-cell maturation antigen (BCMA) can be improved by simultaneous targeting of an additional antigen, e.g., G protein-coupled receptor class-C group-5 member-D (GPRC5D), to prevent BCMA escape-mediated relapse [1]. Another approach is to develop CAR T cells which also secrete a bispecific T-cell engager (BiTE). This was shown in a glioblastoma model in which improved efficacy and reduced toxicity was achieved by the addition of a BiTE against EGFR, an antigen frequently overexpressed in glioblastoma but also in normal tissue, to a CAR specific for EGFRvIII, a glioblastoma-specific tumor antigen [2]. The use of CAR T cells targeting adapter molecules that can be linked to a range of soluble antigen-recognition moieties to enable simultaneous recognition of multiple antigens with a single CAR is another option.

However, a bigger obstacle to use of cell therapy in solid tumors may be the immunosuppressive TME. One approach to overcome this is armored CAR T cells that are co-modified with immunomodulatory agents and that can elicit an endogenous immune response. Preclinical studies showed that treatment with CD19-specific CAR T cells that were modified to secrete interleukin (IL)-12 were able to eradicate established disease in a syngeneic B-cell malignancy model without prior lymphodepletion [3] and a syngeneic model of ovarian peritoneal cancer [4]. IL-18-secreting CAR T cells also significantly increased long-term survival in syngeneic mouse models of both hematological and solid malignancies [5]. IL-18-secreting CAR T cells modulated the TME, inducing expansion of endogenous immune effector cells including endogenous CD8 T cells with a central memory phenotype, macrophages with an M1 phenotype and dendritic cells with a more mature phenotype, and broadened the anti-tumor immune response beyond the CAR target. Another approach is to engineer tumor-targeted CAR T cells to constitutively express the immune-stimulatory molecule CD40 ligand, which displayed superior antitumor efficacy, enhanced recruitment of immune effectors, and mobilized endogenous tumor-recognizing T cells in murine models of leukemia and lymphoma [6].

Finally, CAR T cells can be modified to secrete programmed death (PD)-1-blocking single-chain variable fragments (scFv), which act in both a paracrine and autocrine manner to improve the anti-tumor activity of CAR T cells and bystander tumor-specific T cells in syngeneic and xenogeneic mouse models of PD-ligand (L)1 + hematological and solid tumors [7].

Promising outcomes in CD19 and BCMA CAR T cell trials serve as a proof of principle for this approach to adoptive T cell therapies for cancer. There remain significant limitations to this technology not only in the context of hematologic tumors but even more so in the context of solid tumor malignancies which are immunologically far more complex. However, CAR T cell technology will ultimately become a successful therapy in this context. Additional genetic modification of CAR T cells to generate more potent CAR T cells (i.e., armored CAR T cells) have promise in preclinical studies. Armored approaches have application to approaches based on tumour infiltrating lymphocytes (TILs), T cell receptor (TCR) modified T cells, and CAR NK cells. Rapid translation of these approaches into phase I clinical trials is critical to the further development of CAR T cell technology moving forward.

#### Kunle Odunsi: NO

Cell therapies for solid tumors are being asked to successfully traffic from the blood into solid tumor sites, despite potential T cell chemokine receptor- or tumor-derived chemokine mismatches. Cell therapies need to infiltrate the stromal elements of solid tumors to elicit TAA-specific cytotoxicity, regardless of antigen loss or heterogeneity. Even after successful trafficking and infiltration, T cells become rapidly dysfunctional owing to a hostile TME.

ACT using TILs has demonstrated limited efficacy in advanced melanoma and cervical cancer, with very few patients achieving complete responses [8, 9]. Similarly, in a phase I trial in patients with metastatic non-small cell lung cancer, only two of 13 evaluable patients had complete responses [10]. Most expanded TILs are bystanders and are irrelevant for tumor antigens, with the capacity to recognize autologous tumors limited to approximately 10% of intratumoral CD8 + T cells [11]. Moreover, this approach is associated with a high-risk of adverse events. TIL selection with specificity for mutational neoantigens may be necessary in patients with non-T cell-inflamed tumors but manufacturing remains a hurdle and is currently impractical. The most widespread method of TIL production involving isolation from tumor tissue and expanding in vitro usually takes 6–8 weeks, leading to TIL exhaustion. Moreover, many patients may be unable to wait for treatment involving this delay. Preparing young TILs without selection for antitumor reactivity is much faster but their tumor reactivity is questionable. The immunosuppressive mechanisms in the TME limit the TIL function. Injection of high-dose IL-2 as a standard method to support the growth and activity of injected TILs has several adverse effects.

For CAR T cell approaches, the first question is whether there are suitable target antigens in solid tumors. The ideal TAA needs to be selectively expressed on tumor cells at high levels but not on the surface of important normal tissues (or, if expressed, it should be at a very low level). It should also be expressed on all or almost all of the tumor cells, with success otherwise unlikely. The degree of specificity is critical for safety, with the most feared complication of CAR therapy a catastrophic and rapid ‘on target, off tumor’ event. However, so far, there is no ideal CAR TAA in solid tumors.

Challenges with CAR T therapy includes that cell targets are largely limited to extracellular antigens, e.g., HER-2, PSMA, PSCA, mesothelin, Claudin (CLDN)18.2. The risk of toxicities, complexity of manufacturing, and costs also remain issues. Unsatisfactory clinical outcomes have been observed across solid tumor antigen targets [12]. For example, in a trial of patients with previously treated, CLDN18.2-positive digestive system cancers treated with CLDN18.2 targeted CAR T cells, median progression-free survival (PFS) was only 3.7 months and all patients experienced a grade 3 or higher toxicity [13]. Similarly, responses were limited in a phase 1 trial of castration-resistant, prostate cancer-directed CAR T cells armored with a dominant-negative transforming growth factor-β receptor [14].

While immuno-engineering, such as armored CARs and other approaches, offers the promise to improve CAR-T efficacy, this is yet to be realised. There are no clinical data to support the use of improved CARs with integrated controls, e.g., kill switch, inducible co-stimulation, the delivery of additional payloads, and additional modifications with CRISPR/CAS to ablate immunosuppression.

Only one of 15 evaluable patients had a deep response in the phase 1 SURPASS trial that evaluated ADP-A2M4CD8 SPEAR T-cells co-expressing the CD8α co-receptor with the engineered TCR targeting MAGE-A4 in HLA-A*02-positive patients with advanced cancers expressing MAGE-A4 antigen [15]. In addition, engineering ACTs towards new antigens carries a high-risk of toxicity due to bypassing negative selection in the thymus. In addition, there is increased risk of ‘on-target off-tumor’ toxicity (e.g., T cells targeting carcinoembryonic antigen in gastrointestinal cancers can induce severe transient colitis) [16] and risk of cross-reactivity (e.g., MAGE-A3 with MAGE-A12 and titin cross-reactivity).

Financial toxicity is also a concern, with the cost of CAR T cell therapy out of reach for many patients. Treatment in the US can cost US$375,000 to US$475,000, with the management of treatment-related adverse events and subsequent procedures adding more than US$500,000 to the total cost of the therapy. Access to treatment is thus an issue, especially for underserved minority populations, and patients who are underinsured or uninsured.

Less toxic, resource intense, and expensive alternatives to cell therapies deserve to be further explored. These include immune-mobilising monoclonal T-cell receptors against cancer (ImmTACs), which combine an engineered TCR-based targeting system with a scFv, as well as bi- and tri-specific antibodies, T cell engagers, newer generation immune checkpoint inhibitors, and treatment combinations that provide strategies to overcome tumor immune suppression.

**Fig. 1:**
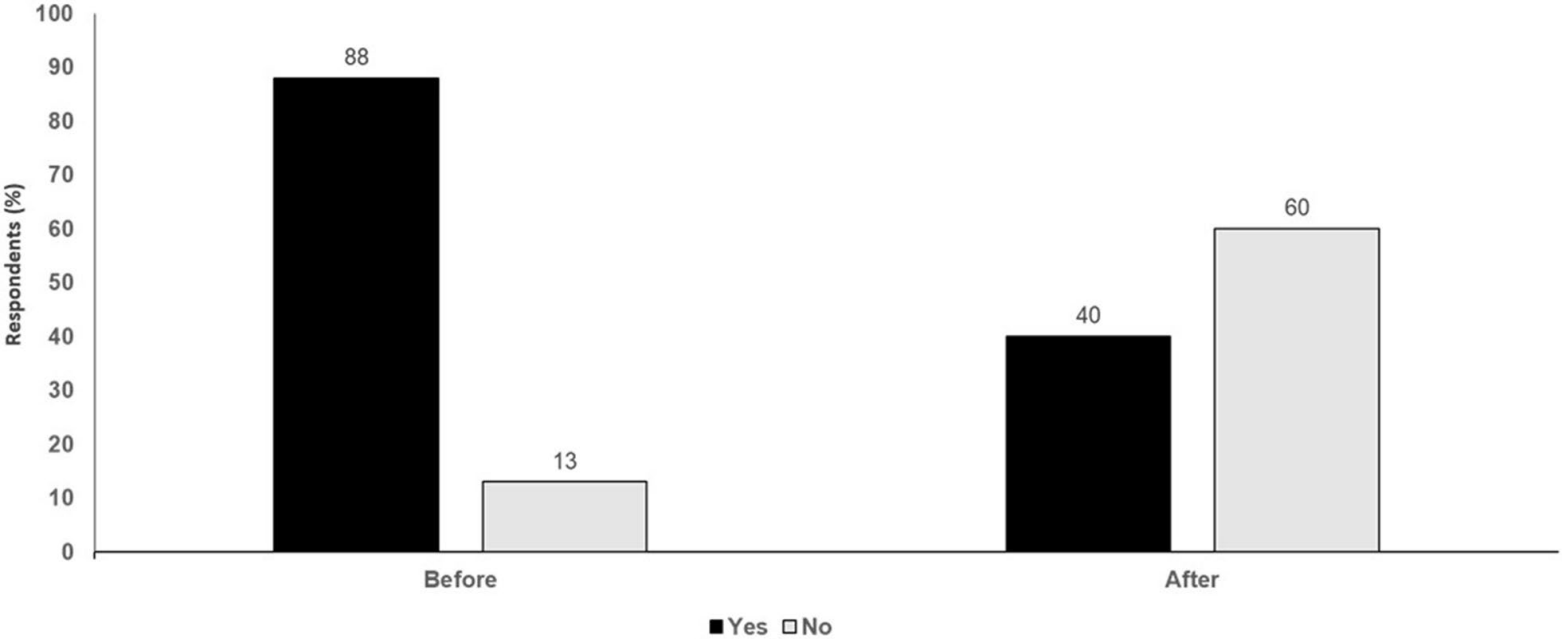
Cell therapy for solid tumor: yes or no? Audience response before and after debate

In conclusion, unselected TIL therapies have limited efficacy while engineering of TCR and CAR T cells poses an inherent risk of both ‘on target, off tumor’ toxicity, and antigen cross-reactivity. There are significant impairments to effective trafficking of ACT to the TME and the promise of newer cellular engineering approaches is yet to be realized. Considering the limited efficacy, associated adverse event, financial toxicity, and the existence of viable alternatives, additional efforts in immunoegnineering and synthetic biology are required before cell therapy for solid tumors become a feasible and effective approach (Table 1, Fig. 1).

**Table 1:** Current challenges with adoptive cell therapy for solid tumor

ACT approach	Challenges
ACT with tumor-infiltrating lymphocytes	Limited success rates
	Most expanded TILs are irrelevant for tumor antigens
	Prepared tumor-specific TILs have questionable tumor antigen specificity
	TIL manufacturing process too long for patients to wait
	Long TIL manufacturing process leads T cell exhaustion
	Immune suppression in the TME limits TIL functions
	High risk of adverse events from treatment and co-administered IL-2
CAR T cells/TCR-T cells	Requires suitable antigen targets that are not often available or adequately tumor-specific
	Limited success across attempts with a variety of tumor antigens
	Limited data on use of improved CARs with integrated controls
	Difficulties in getting cell therapies to successfully traffic into solid tumor sites
	Financial toxicity to the patient due to high costs of therapy, especially for underserved populations and for underinsured or uninsured patients
	Increased risk of ”on-target off-tumor” toxicity

**Key points**

- Current ACT approaches have had limited success in delivering effective tumor-specific responses without off-target toxicities in a cost-effective manner.
- New approaches to improving CARs with integrated controls such as inducible co-stimulation and kill switches, or delivery of additional payloads or modifications to overcome immunosuppression hold promise but have yet to be realized in the clinic.

### Peripheral/blood biomarkers versus TME biomarkers

#### Ryan J. Sullivan: in favour of blood-based biomarkers

Advantages of blood-based biomarker analysis are that blood is more accessible, sampling is safer, and serial sampling is much easier. Blood may be more reflective of the entire disease burden including tumor heterogeneity. It is amenable to analysis by virtually every platform of testing (flow cytometry, enzyme-linked immunosorbent assay, mass spectrometry, nucleic acid sequencing, etc.) and there is ready access to normal samples for comparative analysis in biomarker development. Circulating factors likely represent what is happening in the tumor. These include proteins, exosomes and cell-free DNA in the serum or plasma, peripheral blood leukocytes, other immune cells and circulating tumor cells from the buffy coat, and even red blood cells.

In comparison, tissue-based analysis is the recognised gold standard. The sample is enriched for tumor cells, as opposed to blood which has other shed elements competing with the tumor signal. It is more amenable to nucleic acid sequencing. Moreover, the TME is present and evaluable for physical interaction and investigation (immunohistochemistry, immunofluorescence, spatial transcriptomics, etc.). However, the main disadvantage of tissue-based samples is that biopsies of metastatic lesions are limited in scope for a heterogeneous disease—this sampling bias means that tissue-based analysis is only ideal if you have the entire tumor of every tumor.

Ultimately, the choice between blood or tissue biomarker may depend on the intended strategic goal. One strategy is biomarker enrichment, which is the current approach with tumor mutational burden (TMB) and high microsatellite instability/mismatch repair deficiency, both of which can be done with tissue or blood, or PD-L1 testing, which requires tissue for staining. The aim is to identify immunotherapy-responsive patients for treatment selection and/or enrolment to a clinical trial based on biomarker status. Either tissue or blood-based analysis are possible options in this scenario. However, tissue-based analysis is often associated with underwhelming predictive capabilities. For example, data from the CheckMate 067 trial showed that the level of tumor PD-L1 expression alone is a poor predictive biomarker of overall survival (OS) in patients with advanced melanoma treated with nivolumab plus ipilimumab or with nivolumab alone [17]. Analysis of immune- and tumor-expressed genes by whole-exome and bulk RNA sequencing of tumors in patients with melanoma treated with an immune checkpoint inhibitor also generally showed poor predictive ability of response and survival [18]. Conversely, serum proteomic analysis may be predictive of immune checkpoint inhibitor response/non-response in melanoma. Whole plasma proteomic profiling of patients with melanoma treated with anti-PD-1 therapy revealed differentially expressed proteins between responders and non-responders that may enable a liquid biopsy to predict anti-PD-1 response [19].

A second strategy is biomarker-directed escalation, in which a serial biomarker assay is performed before the start of treatment and again during therapy, with continued treatment in immunotherapy-responsive patients and escalation of treatment in patients who are non-responsive. There are limited data to support this serial biopsy approach in tissue other than with neoadjuvant therapy. However, a molecular signature of circulating tumor cells in patients with melanoma can be used to quantify early tumor response using blood-based monitoring [20]. Although baseline data were not predictive, a decrease in circulating tumor cells within 7 weeks of therapy correlated with improved PFS and OS. This suggests an early on-treatment liquid biopsy may be a feasible approach, with serial analysis favoring a blood-based approach.

The final scenario is termed next level biomarker optimization, in which a biomarker assay can be used to predict immunotoxicity. This may involve either biomarker enrichment or biomarker-directed escalation but only a blood-based approach would be feasible.

In conclusion, there are some scenarios where tissue-based analysis would be optimal if there is limited tumor heterogeneity or maximal tissue (i.e., in a neoadjuvant setting). However, in most scenarios, blood-based biomarkers allow safe serial analysis, provide better representation of the entire tumor burden and its heterogeneity, may help provide insight into immune-tumor interactions, and are the only approach that may predict toxicity.

#### Samir N. Khleif: in favour of tumor-based biomarkers

In a review of cancer vaccine trials, although activation of T cells and immune response was often reported in the periphery, patients typically did not respond to treatment [21]. Thus, the presence of activated, tumor-specific T cells in the blood does not mean they are active in the tumor. It is what happens in the complex TME that is important, and not what the periphery might tell us. This may explain why every biomarker that has been approved and/or shown a correlation with response is tumor-based. PD-L1 expression in pre-treatment tumor biopsy samples has been shown to be associated with response rate, PFS, and OS [22]. Pan-tumor genomic biomarkers, such as TMB and T cell-inflamed gene expression profile, also predict response to PD-1 therapy [23]. TMB is an independent predictor of response to treatment with various immunotherapies across diverse cancers, again indicating that it is what happens in the tumor that is most important [24].

Indeed, tumor heterogeneity is an important consideration. However, this is yet another factor why the peripheral immune response may not be of great value since it will not reflect the essence of its interaction within the milieu of the heterogeneous tumor. Accordingly, the TME would have the ability to take into account the more complex picture of the immune response and its effect on the outcome. A case in point, TME Immunoscore, which reflects the complex multi-factorial immune response is clearly predictive of survival [25]. High Immunoscore is also predictive of response to anti-PD-1/L1 therapy, as are tumor biomarkers identified by multispectral imaging and mapping [26].

**Fig. 2:**
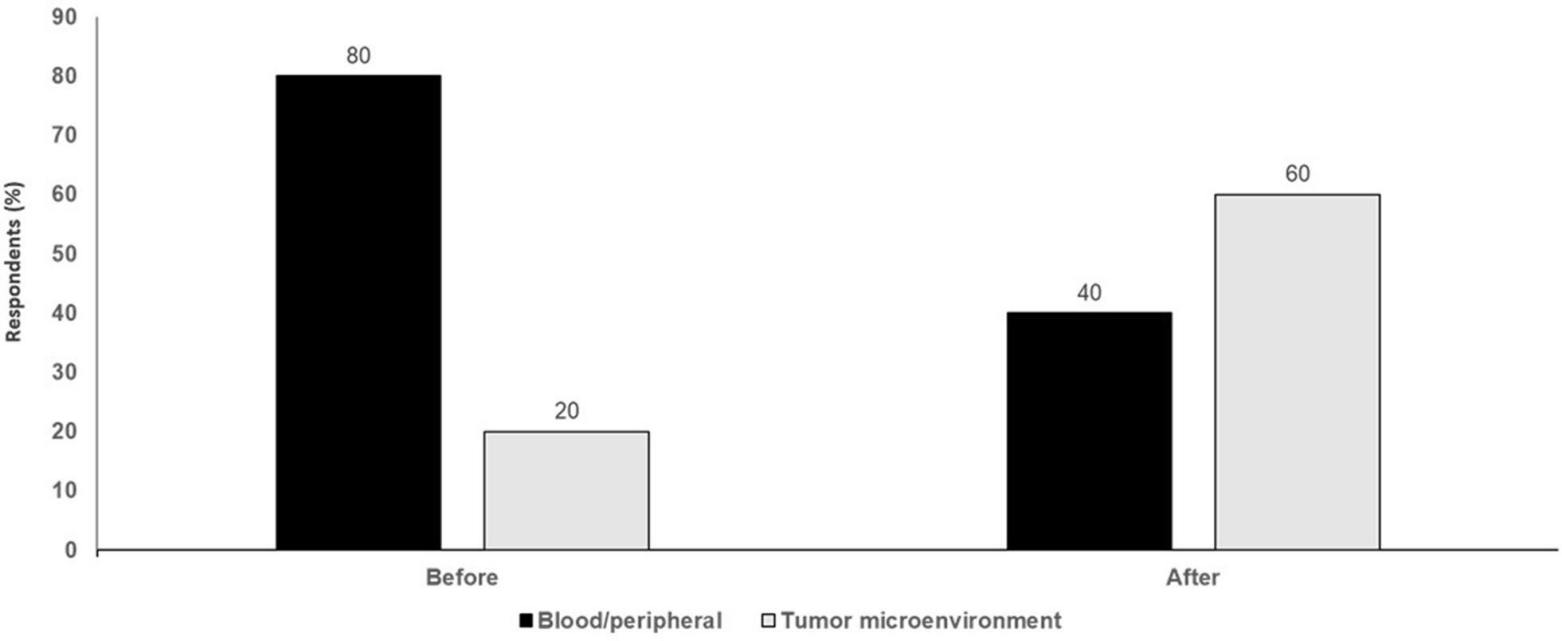
Peripheral/blood biomarkers versus TME biomarkers. Audience response before and after debate

Even in the context of cold tumors, which are characterized by the lack of T-cell infiltration, the underlying mechanisms, e.g., impaired T-cell priming and deficient T-cell homing to tumor beds, are based in the TME [27]. As such, it is understanding the processes occurring in the TME that is important. Biomarkers can be prognostic, predictive, response/mechanistic or outcome-focused and tumor-based biopsy samples can address all of these scenarios since it reflects what is happening in the tumor. However, it is not a case of either or, but rather it should be both; there is a large number of potential biomarkers of different immune variables used to predict immune checkpoint blockade responses and/or patient prognosis and these can be either tissue or blood-based. Blood-based biomarkers can be helpful, but clearly tissue-based biomarkers are currently more important (Fig. 2).

**Key points**

- Blood-based biomarker analysis offers the advantages of safer sampling, especially serial sampling, it may be more reflective of tumor heterogeneity, and it can be analysed using a wide range of platforms.
- However, blood-based biomarkers may not be truly reflective of the complex TME every biomarker that has been approved and/or shown a correlation with response is tumor-based.
- Ultimately, it should not be a case of either or, but rather it should be both the choice between blood or tissue biomarker may depend on the intended strategic goal.

### CAR T cell therapy versus CAR natural killer cell therapy

#### Marco Ruella: in favour of CAR T cell therapy

There are now six approved CAR T cell products for multiple CD19-positive and BCMA-positive hematological malignancy indications. However, there are no approved or close to approval NK CAR products [28].

The ultimate goal of the immune system is to activate T cells against cancer cells. In the traditional model, the innate immune response that includes NK cells, occurs early on with its role in part to prepare for the more significant adaptive T and B cell response. NK cells have a half-life of only around 15 days in peripheral blood, whereas the half-life of T cells is 30–160 days with T cell memory persisting for 8–15 years [29]. Although some form of NK memory is now thought to exist, the adaptive immune response is more important in targeting cancer cells and explains why T cells have been the primary focus of CAR technology [30].

An important feature of CAR T therapy is its potent expansion and prolonged persistence [31]. For example, persistence of tisagenlecleucel in the blood of pediatric and young adult patients with CD19-positive relapsed or refractory B-cell acute lymphoblastic leukemia was observed for up to 20 months [32]. High rates of durable responses have also been shown with tisagenlecleucel in adults with relapsed or refractory diffuse large B-cell lymphoma or follicular lymphoma [33, 34].

However, despite the remarkable results achieved with CAR T cell therapy, there is still a subset of patients that do not initially respond or that eventually relapse. The failure of CAR T cell treatment in these patients may be due to an exhausted T cell phenotype with lower persistence and antitumoral activity [35]. This has led to the search for novel approaches to overcome this failure, e.g., by changing the effector cell. NK cells that have been engineered to express a CAR are candidate effectors. Unlike CAR T, CAR NK cells can recognise the antigen via other receptors in addition to the CAR. However, to date there are very limited data for CAR NK therapy. In a trial of 11 patients with relapsed or refractory CD19-positive non-Hodgkin's lymphoma or chronic lymphocytic leukemia, eight had a response to treatment with CAR NK cells [36]. However, many of these patients also received other treatments, including hematopoietic stem cell transplant or rituximab. Only one patient had a complete response at 1 year without other treatment. In comparison, 5 year PFS rate in patients with diffuse large B-cell lymphoma treated with tisagenlecleucel was 31% [37]. Moreover, persistence of CD4-positive CAR T cells has been shown for up to 10 years in patients with chronic lymphocytic leukaemia [38], while persistence of CAR NK cells has only been shown at a low-level for 1 year [36]. As part of innate immunity, NK cells may lack proliferation and persistence and, in theory, do not establish memory. In addition to persistence, cells need long-term functionality. B-cell aplasia and CD19-loss are markers of functional persistence and indicate the long-term function of CAR T cells.

To conclude, both approaches are interesting but are at different stages of development. Six CAR T products are approved for many indications with thousands of successfully treated patients whereas data have only been reported for 11 CAR NK treated patients with heterogeneous diagnoses and the use of other treatments.

#### Katayoun Rezvani: in favour of CAR natural killer cell therapy

CAR T cell therapy has resulted in a paradigm shift in how patients are treated and is fast becoming a mainstay of therapy for hematological malignancies. However, to date, all FDA-approved CAR T cell products are autologous and their manufacture and costs can be issues. Other significant limitations include the intrinsic poor quality of T cells derived from patients with cancer, tumor antigen escape, and CAR T related toxicities, in particular cytokine release syndrome (CRS) and immune effector cell-associated neurotoxicity syndrome (ICANS).

NK cells are part of the innate immune systems and have intrinsic activity against many cancers, e.g., glioblastoma, acute myeloid leukemia, multiple myeloma, etc. There is no or low risk of graft-versus-host disease with NK cells and antigen recognition takes place through a complex array of activating and inhibitory receptors that are endogenously expressed on NK cells.

Around 30–50% of relapses after CAR T cell therapy are due to target antigen loss. This is a major problem given that autologous T cells are only able to recognise the target via CAR-directed antigen recognition. However, in NK cells, the integration of inhibitory and activating NK cell receptor signals regulates the NK cell’s decision to kill. In normal cells, the inhibitory signals triggered by KIR-HLA-I molecule engagement overrides any activating signals and prevents cytotoxicity. In the context of cancer, expression of stress ligands, recognized by NK cell activating receptors, in conjunction with low expression of HLA-I molecules which attenuates the triggering of inhibitory receptors, results in an activating signal for NK cells to kill. Thus, CAR NK cells can mediate killing both via the CAR and via their innate receptors, meaning relapse through target antigen loss may be less critical after CAR NK cell therapy.

The intrinsic quality of CAR T cells have been shown to have a profound effect on the likelihood of response. Heterogeneity in the cellular and molecular features of CAR T cell infusion products contributes to variations in efficacy and toxicity, with T-cell exhaustion associated with a poor response [39, 40]. Manufacturing failures due to the poor quality of the starting material or pre-existing lymphopenia are also recognized limitations. There is also the potential for infusion of contaminating tumor cells, as shown by the unintentional introduction of an aberrantly expressed anti-CD19 CAR into a single leukemic B cell during T cell manufacturing [41]. With NK cells, the source can be from any healthy individual, e.g., peripheral blood mononuclear cells, umbilical cord blood, induced pluripotent stem cells (iPSCs) or hematopoietic stem cell cells, and so removes the problem of the intrinsic quality of the cells. Off-the-shelf engineered NK cell lines such as NK-92 cells have also been explored in the clinic and offer a homogeneous product that is easy to manipulate and engineer, with high proliferative capacity. However, because NK-92 cells are derived from a patient with NK lymphoma, they need to be irradiated which may result in limited in vivo persistence. The use of induced pluripotent stem cells also offers a potentially unlimited source of NK cells for therapy and iPSC-derived CAR NK cells are being tested in multiple settings in the clinic. Our group is interested in exploring umbilical cord blood as a rich source of NK cells for immunotherapy. In a first-in-human clinical trial, we reported the safety and promising activity of CB-derived CAR19/IL-15 engineered NK cells in patients with lymphoid malignancies [36].

Currently the costs for approved CAR T cell products in the USA are in the region of US$ 375000–475000 per dose and each product can only be used in a single patient, i.e., there is no opportunity for scalability to help reduce costs. For many patients, even in wealthy developed countries, the high cost of CAR T cell manufacture precludes access. In contrast, NK cells can be developed from various sources and stored as a truly off-the-shelf product, thereby reducing cost and increasing accessibility. As mentioned earlier, both NK cells lines and iPSC NK cells provide a potentially unlimited source of NK cells for immunotherapy. We have also shown that we can manufacture multiple doses of CAR-NK cells from one umbilical cord blood unit. In an ongoing phase I/II clinical trial evaluating the safety and efficacy of CD70 CAR NK cells for cancer immunotherapy, we have manufactured and cryopreserved over 100 patient doses from one cord blood unit, significantly reducing the cost of manufacturing and therapy. Similarly, in a phase I/II study for glioblastoma, we have successfully scaled-up the manufacturing of multiplex CRISPR gene-edited *NR3C1*/*TGFBR2* double KO CB-NK cells, with 120 patient doses manufactured and frozen from one cord blood unit.

CAR T cells are associated with certain toxicities, in particular CRS and neurotoxicity, which thankfully are associated with low mortality, but may still require intensive care in 30–50% of patients with high associated financial costs. These toxicities are not observed with CAR NK cells, although the reasons for this are not fully understood [36].

**Fig. 3:**
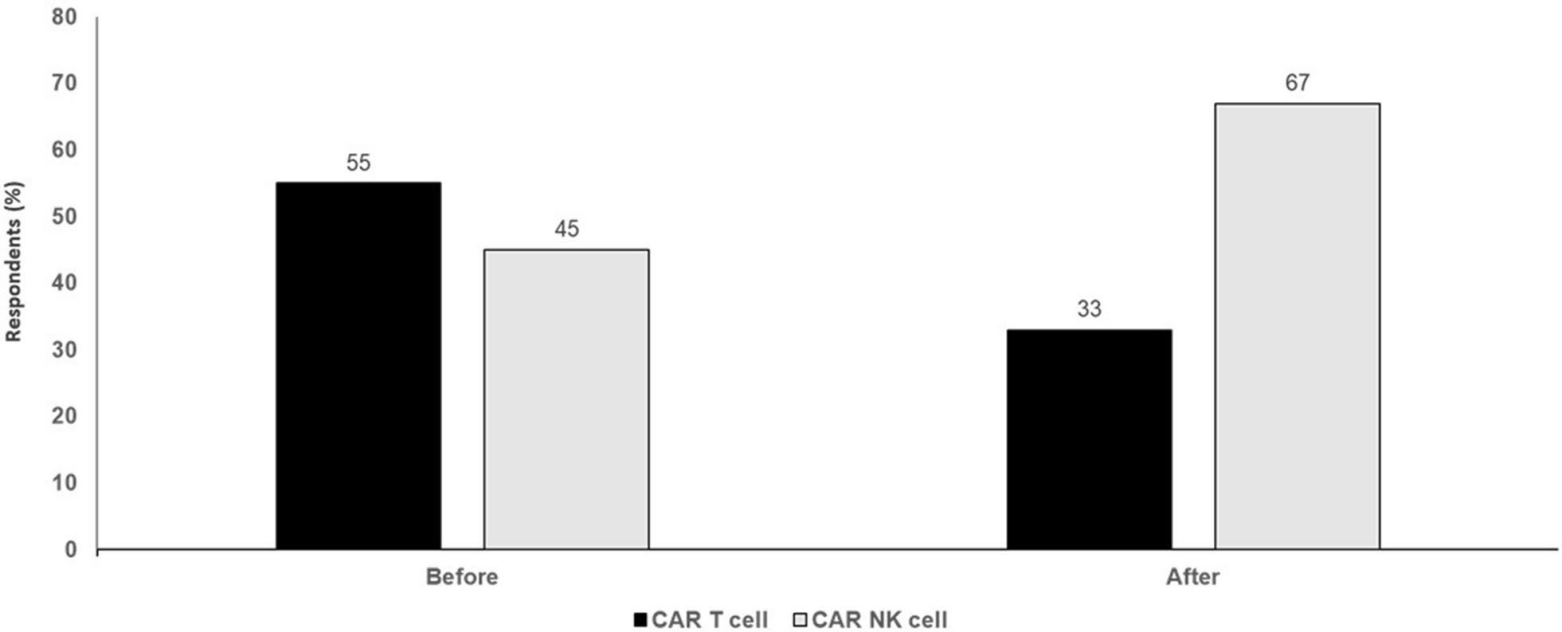
CAR T cell therapy versus CAR natural killer cell therapy. Audience response before and after debate

It is important to acknowledge the limitations to CAR NK therapy. NK cells have a short half-life, with limited persistence in the absence of cytokine engineering or exogenous cytokine support. The best source of NK cells for CAR engineering is not yet clear. CAR NK cell potency can still be improved, e.g., by increasing tumor infiltration and/or overcoming tumor suppression and escape. The optimal costimulatory molecule and signalling endodomains for an NK cell CAR construct are not yet known. Challenges with cryopreservation also remain, as NK cells are more difficult to freeze than T cells, with loss of in vivo potency post-thaw. Finally, there is clearly less clinical data and shorter follow-up for patients treated with CAR NK cells than is available for CAR T cell therapy. Nonetheless, CAR NK cells may represent the next paradigm shift in ACT, with the promise of greater efficacy, less toxicity and a more cost-effective option that will allow more patients to access treatment (Fig. 3).

**Key points**

- Despite the successes of CAR T cell therapy, there is still a subset of patients that do not initially respond or that eventually relapse.
- CAR NK cells can recognise the tumor antigen via receptors other than the CAR and offer the potential of greater efficacy, less toxicity and a more cost-effective option allowing more patients to access treatment.
- However, to date, evidence to support CSR NK cell therapy is limited and several challenges remain e.g. limited persistence in the absence of cytokine engineering or exogenous cytokine suppor.

## Conclusions

The Immunotherapy Bridge Great Debate included the presentation of counterpoint views from leading experts on contemporary clinical issues. Presentations were not intended as a rigorous and/or systematic assessment of the field but instead allowed the opportunity to highlight some important questions and current controversies. These debates are obviously more nuanced than the simple for or against/yes or no format encourages; however, it is hoped that these discussions can help focus attention on these issues, stimulating further research needed to improve our understanding of different therapeutic approaches.

## Data Availability

Not applicable.

## Abbreviations

ACT: Adoptive cell therapy
BCMA: B-cell maturation antigen
BiTE: Bispecific T-cell engager
CAR: Chimeric antigen receptor
CRS: Cytokine release syndrome
ICANS: Immune effector cell-associated neurotoxicity syndrome
IL: Interleukin
IPSCs: Induced pluripotent stem cells
NK: Natural killer
OS: Overall survival
PD-1: Programmed death-1
PD-L1: Programmed death ligand-1
PFS: Progression-free survival
SCFV: Single-chain variable fragments
TAA: Tumor-associated antigens
TCR: T cell receptor
TIL: Tumor-infiltrating lymphocyte
TMB: Tumor mutational burden
TME: Tumor microenvironment
